# Observations on the Zanthoxylon

**Published:** 1802-11-01

**Authors:** G. Bellamy

**Affiliations:** Oreston


### *Observations on the Zanthoxylon; communicated by* Dr. G. Bellamy, *of Oreston.*

THE following obſervations are intended to illuſtrate the powerful and good effects of a medicine very little known in this country, but which appears to me, from my own experience in the uſe of it, and what I have heard from that of others, to bid fair for leſſening an opprobrium of the art, namely, inveterate ulcers of the legs, without a conſtitutional affection. I do not mean to confine its operation to the leg, as it will doubtleſs be equally or more efficacious in other parts of the body; but inveterate ulcers, not dependent on the ſtate of the ſyſtem at large, being very rare, except on the leg, its uſe on other parts will require the aid of conſtitutional remedies, or it may prove only palliative; even ſo far it is a deſideratum. The remedy is the Zanthoxylon, commonly called in the Weſt Indies Hercules' Club. There is a paper or two already in your Journal about it, communicated from Jamaica. I hope, what I have to ſay, will add to the knowledge of its general properties, and of its ſingular efficacy in ulcers; I ſay ſingular, becauſe it ſucceeded where every thing elſe that my information, or that of my medical friends near me, could ſuggeſt, had failed. It was the powder of its bark which I applied to the ſurface of the ſore, a piece of dry lint over, and if any, a ſlack bandage, or proportionably tight, according to the degree of retroceſſion of the ſurrounding integuments, and relaxed ſtate of the muſcles; preferring rather a generous diet with vegetables, and no ſalt meat But I am told by Dr. Leonard Gilleſpie, late of Port Royal Hoſpital, Martinique, who firſt ſupplied me with the article, and directions for its uſe, that a tincture of the ſame locally applied is very efficacious; but to this form it may be objected, that we do not know how much we may owe to the ſtimulus of the ſpirit. Dr. G. further informed me, that employed internally, it proved according to the doſe, tonic, diaphoretic, and diuretic; and is uſed alſo as a febrifuge; it is aſtringent and bitter. To my brother officers in the navy, who know how many lives have been loſt, and rendered uſeleſs to the ſervice, (and nearly ſo to Society at large, in too many inſtances a burthen to it and themſelves) any improvement in this loathſome part of ſurgery muſt be particularly intereſting; in all ſhips of war it bears a great proportion in the ſick liſts, and has exiſted with perhaps unprecedented malignity in the late war; in the Mediterranean, at the Weſt Indies, and even in the Channel. However, I do not mean to infer that the Zanthoxylon will avail in that malignant ſtate of ulcer, (which is ſcarce ever to be ſeen on ſhore except in our naval hoſpitals) which there is but too good grounds to think contagious; infectious they certainly are, ſo much ſo that it has been plainly proved, that every ulcer patient muſt be furniſhed with a diſtinct ſponge, bandages, &c. &c.; for thoſe which have been uſed for a malignant ulcer cannot be employed with impunity on a ſimple inciſion or mere ſcratch. For the uſe of Zanthoxylon in this dreadful diſeaſe my experience is not ſufficient; but this I will be bound to aſſert, that their gangrenous ſloughs once removed, and the ſloughing proceſs, which appears to me their chief characteriſtic, corrected, the Zanthoxylon will haſten the final cleanſing, and rapidly promote the healing action; but until that is done, we might as ſoon expect it to cure where there is a diſeaſed bone beneath, or a venereal, ſcrophulous, or other habit. Again, how far it may prevent the malignant ſtage, by being early employed in the incipient ſtate of ulceration, and in moſt common ulcers, (for moſt wounds on board of ſhip do ulcerate, and even thoſe malignant ones will be found to have been but ſimple wounds or ulcers) till an abſorption of the ſpecific virus by ſome unfortunate and perhaps unheeded communication: How elſe are we to account for the total abſence of the malignant ulcer in many ſhips, though on the ſame ſtation, and expoſed to all the ſame relative circumſtances, till ſome miſerable object conſuming under it, has been ſent on board for a paſſage to an hoſpital, &c. See then what vigilance is neceſſary! and how much better it would have been to prevent than to cure, alas, than to endeavour to cure *only*.

It

It will be plainly feen, when I fhall have related my cafe of the efficacy of Zanthoxylon, that that was not a cafe of malignant infectious ulcer, at leaft of the kind above mentioned; perhaps, it may be called an ulcer, fui generis. Singular in its nature to me, and equally furprifing to many who faw it. Should it be afked why did I keep the man fo long on board, I anfwer he was not only willing to ftay, but unwilling to go to an hofpital, till he did go; and then required incentives, or would have been eafily induced to ftay, could I have flattered him any longer. My officers were pleafed with my earneftnefs; I fhould have been lefs wife, I hope I may be able hereafter to add, and the public lefs profited. Thefe obfervations were at firft intended for the Honourable Commiffioners for Sick and Wounded Seamen; they have them it is true in my Journal, but in a more diffufed ftate. The war being over, I think they will be rendered more public by means of your work, to the furgeons of the Navy and to the Profeffion at large. But to the Medical Board, at this time, they might not be fo opportune. Moreover, I underftand the late Dr. Johnftone had received a fample of the Zanthoxylon, with a much better account than I am able to furnifh.

James Pomeroy, feaman, about thirty years of age, by trade a fail-maker, and ufually employed as fuch on board, on the 4th of Auguft, 1800, came to his Majefty's fhip Spencer from the Aquilon, juft arrived from the Weft Indies. I found him afflicted with feveral fmall, ill-conditioned ulcers on the foles and fides of the feet, and on one great toe. He told me he had been thus afflicted for feveral months, though occafionally better; he had fome fufpicion that jiggers were the exciting caufe of the firft fore, but that others had taken place from time to time, in form of a fmall bluifh coloured veficle; which thofe who are experienced in true fcurvy will correctly form an idea of, and may be beft compared to vibices. It may be neceffary to inform fome, that the jigger is a fmall infect of the tropical climate, which thofe who go bare foot are very liable to get infinuated, gradually, very deep in the feet, forming to itfelf a nidus, creating a moft painful fore; for the cure of which, the removal of the animal is firft neceffary, and at this the blacks are very expert. Admitting fuch to have been the occafional caufe, it was of very little confequence to confider that at prefent. On the whole, he had very gradually got into a deplorable ftate, fuffering conftant pain, quite unable to ftand, and his health undermined. From the appearance of the wounds, his general health and countenance, the nature and intolerable fœtor of the difcharge, I confidered the cafe to be completely fcorbutic, butic. Accordingly, I gave him lemon juice, and applied lint compreſſes immerſed in the ſame acid twice a day to the ſores. This was continued till the 13th, when he took four ounces of the acid, with the ſame quantity of a mixture of bark, and repeated the dreſſings as before, till the 18th; he then took eight ounces of each till the 20th, when he omitted the bark, becauſe it purged him; but the acid was repeated in both ways, and I gave an opiate till the 27th, when pulv. cinchon. was applied to the ſores, and acid repeated as before.

Rep. ad 17th Sept. when all the ulcers began to ſkin over, and that very rapidly; very ſimilar to the ſkinning proceſs (very different from cicatrization), of a chancre, after the morbific action is deſtroyed by a cauſtic. R. Succ. limon. ℥xij. quotidie, et applic. pulv. cinchon. ulceribus. Antiſcorbutic diet.

R. ad 10th October, when I omitted all antiſcorbutics, and gave calomel, gr. ij. quotidie; applied ſimple dreſſings, and gave opium occaſionally, becauſe the ulcers were again at a ſtand, and very painful.

17th. Ulcers worſe; ſpread again, and very painful; feels weak from the calomel, which I omitted, and reſumed the former plan, dreſſing with powdered bark on the ſurface, and dry lint over; and taking a pint of lemon juice a day, till Jan. 4, 1801, when I dreſſed one foot with hydrarg. nitrat. and the other with bark.

7th. Omitted lemon juice, becauſe he had it, in form of a ſherbet, like the ſhip's company, with plenty of vegetables. But when lemon juice was preſcribed, it is to be underſtood, he took whatever quantity is expreſſed, unmixed; not exceeding half a pint for a doſe; and that he uſed to drink off with as much eaſe as water; and, I may add, with as little permanent benefit.

30th. R. u. a. et ſucc. lim. ℥iv quotidie. A more ſcorbutic appearance. Repeated to the middle of February, when one foot was healed, and continued well to 17th March; but the other gets a repeated ſloughing, or hardened collection of matter and dreſſings; on the removal of which, the ſores are found nearly as before. Dreſs with a ſolution of blue vitriol.

March the 18th. A ſcabby eruption, for ſome days, on the ſide of his noſe, not unlike herpetic ſcab, or rather like lepra; being whitiſh, brown, and incruſted; ſuch, he ſays, he has been accuſtomed to in warm weather. If he was not ſo poſitive of never having had the venereal diſeaſe, and did not mercury aggravate the ulcers, ſhould determine ſuch to be venereal; alſo round a healed ſore of one foot, ſome white ſcales come out firſt in form of tetter.

April

April the 10th. One foot continues quite well, the other nearly ftationary; to affift which, applied adhefive ftraps, and continued the fame general tonic method to the 13th of May, when, for the firft time, I applied pulv. cortic. Zanthoxylon twice a day to the furface of the fores, and over it dry lint. 18th. Ulcers certainly improved they contract and dry, without granulation, as the others did, after the manner of chancre. 20th. One ulcer that lately broke out is well again, the other (for they are now reduced to one) has a florid, folid, and healthy appearance, which it never has had before. R. pulv. zanthox. N. B. He has not taken lemon juice, or other medicine, fince he began the Zanthox. fo that its action is totally unaffifted, except by a fimple nutritious diet; and that he has never been without; he generally had frefh beef every day, plenty of vegetables, and always a pint of wine, tea, fago, chocolate, &c. 24th. Much better, drying, hard and fair, but by contraction has caufed a painful tightnefs of furrrounding integuments, with fome inflammation. R. ad 31, when not near fo well; the florid-coloured granulations, or rather furface, has difappeared; difcharge thin, and acrid; much pain, furrounding rednefs, and fenfibility. Omit the Zanthox.; apply fimple dreffing till the 3d of June, when he went to Plymouth Hofpital. Mr. Fuge, firft furgeon of that eftablifhment, has favored me with an account of what was done for him there, though without avail, till recourfe was had again to the Zanthox. with the quick, powerful, and permanently good effects of which he was very agreeably furprized, and was decifive, in his opinion, that he owed his cure to the operation of that medicine, which it performed in three or four dreffings; not by a gradual formation of cicatrix, after being brought to a level by granulations, but by cleanfing and coming to a fmooth red furface, very much like the look of a part from which you have recently removed the cuticle after a phlegmon, and healing by a procefs of fkinning, like recent blifter or chancre.

Whilft in the hofpital he had a little of that encruftation on one noftril, of which I have before fpoken; it was always trifling in its appearance, and without pain or inconvenience; was never complained of by him, and might have paffed unnoticed by any one who was not interefted in feeking for any probable developement of the caufe and nature of his infirmity; nothing was ever applied to it. Of the fame apparent infignificance, was a little interruption of fpeech, fomething like lifping and faltering of the tongue; this I had thought little of, except as an organic fault, or as the effect of habit; but there is reafon to think it a degree of difeafe, from the circumftance of a partial paralyfis of one arm, which came on fuddenly a week week or fo after the healing of the wound; and the impediment of fpeech increafed at the fame time. By ftimulants, however, he got the better of thefe complaints, fo far as to be difcharged from the hofpital, after being in it about three months; not able to walk, or ftand firm, without the aid of crutches; which is not to be wondered at when we confider the pernicious effects of inaction on the fyftem at large, which was very much enervated, and on the lower extremities in particular, which he had not ufed for near eighteen months, generally going about on his hands and knees, or podex.

The mufcles of the legs (tho' well formed, as was his frame in general) were quite flabby, the ancle joints fo weakened, and he had fo little command of the flexor and extenfor tendons, that on lifting the feet from a point of fupport, they would tremble inceffantly, and very quick, which he had not the leaft power to reftrain; they fhook as if they were hung on wires; this being confidered, it is more to be wondered at he got on fo faft. He then being defirous of going to his friends, Mr. Fuge very kindly promoted his being invalided. His wound remained well, and he was recovering, though flowly, the ufe of his limbs, and a reftoration of tone in the fyftem, which had been fo long, as alfo his appetite, delicate and weak, more like a fafhionable lady than a failor. Neither at the time of his paralytic affection, nor fince, have I had an opportunity of queftioning him relative to his imperfection of fpeech, which it may be neceffary to obferve was not always alike, and was much worfe, two or three times, when he had taken a glafs of wine extraordinary. I dwell upon thefe circumftances, to fhew the atony of the fyftem; and the fitnefs of the method of cure, by tonics and ftimulants, proved to be adapted to his general health, and to the particular atonic ftate of the ulcers and the morbid action fo long entertained there; if that can be confidered action, whofe very effence confifted in inaction, confirmed by long habit, which the whole train of irritants was unable to roufe, or alter for the better. Though I could give a great deal of pain by fuch means, and enlarge the wound by floughings and efchars, I never could produce inflammation, correctly fpeaking, nor that healthy degree of it fo effential in fuch cafes, until I applied the Zanthoxylon. Such action, it is to be obferved, had been induced a few days before I fent him to the Hofpital, though it again took an unfavourable turn, which, with all circumftances confidered, and the expectation of the fhip going abroad, made me decide on removing him; though it is more than probable I fhould foon have fucceeded in his cure cure on board, yet it may be right to attribute ſomething to change of place, mode of life, and ſtate of mind.

It may be alleged, I ſucceeded in curing ſeveral of the ſmaller ulcers, and one foot entirely; but the ſmaller ones were of conſiderable leſs duration; the one which remained was of the longeſt, was larger, deeper, worſe conditioned, and worſt ſituated; in the centre of the ſole, and according to a law which frequently applies, the action which was before divided, appeared to be here collected; it was occaſionally much worſe, but never better; till at the laſt, than before the healing of the others; beſides as it held out the longeſt, the force of habit may alſo explain the inefficacy of long continued means; and though as in diſeaſes of ſome characters, you preſerve the principle of action, you vary the agents; as we often find a good account in changing from bark, to bitters, ſteel, &c. All the uſual means had failed; they had had a long and fair trial, and though I will not go ſo far as to ſay a cure could not have been obtained by perſevering in them, or that ſomething elſe might not have been found efficacious; yet it is plain that ſomething new was deſirable; happily for the patient, and ſatisfactory to me, therefore was the deſideratum found in the Zanthoxylon; which beſides its general qualities, I ſee no greater difficulty in admitting as a ſpecific, in certain caſes of ulcer, than to admit bark for intermittents, or mercury for lues; if it is not proved that they are the ſine qua non, perhaps it may be admitted that they are the beſt, and that they are ſufficient antidotes. I may be aſked, What are thoſe certain caſes of ulcer? I am puzzled to deſcribe even the one in queſtion. It was never larger than a ſhilling, for months not bigger than a ſixpence; about the eighth of an inch deep, thin ragged edges, like a margin of black ſkin falling down on the ſurface, as often renewed as removed; ſurface honeycombed, livid, with a thin, acrid, fœtid, diſcharge; at other times nearly filled up, fair, almoſt dry, gathering a ſcab, which was renewed as often as removed; diſcloſing the ſore exactly in the ſame ſtate every day, for a month or two. But I think the beſt indication for the uſe of the medicine is, in all caſes, where the uſual practice has failed, where there is no diſeaſe of bone, or any thing acting as an extraneous body; nor any virus in the ſyſtem. Even in theſe caſes, though it cannot effect a cure, it may anſwer many good purpoſes, and appears worthy of extenſive experiment.

This treatment in the hoſpital conſiſted, firſt, in a liberal uſe of bark, with porter, and nutritious diet, and opium occaſionally. 6th June, he had boluſes of calomel and antimony. 7th, Having a ſlight attack of fever, he had a febrifuge mixture, with camphor, &c. with porter, wine, and a pill of calomel.

calomel, gr. iij. and opium gr. ij. which was continued to the 12th, with the addition on the 10th, of a ſtrong decoction of bark, ℔ſs. in which ℥ſs of ſtrained opium was diſſolved: this was uſed as a lotion to the ulcer; the camphorated mixture and opium were reſumed till the 15th. — 16th he began a free uſe of bark and opium till the 17th July, with porter, wine, &c.; then a liniment of linim. ſapon ℥iij. and tinct. cantharid. ʒj was applied to the margin of the ulcer, and the former treatment of bark, &c. repeated to the 1ſt of Auguſt, when a bliſter was applied near the ſeat of the ulcer, which was repeated on the 9th, bark, opium, wine, and porter being continued to the 16th, when he took a mixture of pulv. and tinct. cinchon. and the tinct. canth. was locally repeated. Purgatives had been occaſionally adminiſtered, and as yet not the leaſt ſalutary change induced; the ulcer larger, ſloughy, and without action, though exceſſively painful: a ſolution of argent. nitrat. was then applied, with no other effect than producing a large eſchar, which was repeatedly produced, but no diſpoſition to ſalutary action induced; nearly as if it had acted on dead matter: then ſucceeded the yeaſt poultice, which cleanſed off the remains of ſloughs, but left a ragged, dark-coloured ulcer, on which an ointment with hydrarg. nit. did nothing. I then mentioned to Mr. Fuge the flattering character I had received of the Zanthoxylon in obſtinate ulcers in particular; and the good effect, though tranſient, it had had on Pomeroy, juſt before I ſent him on ſhore; as alſo in cleanſing ſeveral other ulcers, and partly diminiſhing their ſize, though incapable of cure by ſuch means, from Necroſis; which were ſent home, under my care, in the ſame voyage from the Weſt Indies. Mr. Fuge had read of the virtues of the medicine, and met my propoſal for its trial in a very friendly manner: I accordingly ſupplied him with ſome of the bark, which was applied under his immediate inſpection; and to his great ſurpriſe, poſitively performed a cure by covering the ſurface of the ulcer three or four times with the powder of the ſame; and without any other aſſiſtance. I mentioned to Mr. Fuge my intention of publiſhing the caſe, which he joined with me in thinking worthy of public notice, and very politely furniſhed me with the preceding account of his hoſpital treatment. Why it did not cure him when uſed on board might be owing to my ſuſpending its uſe too ſoon, on account of its powerful excitement, and the inflammation it induced: perhaps it was even right to omit its uſe for a few days for that reaſon; and I doubt not, had I then reſumed its application, he would have been cured; but all circumſtances conſidered, I thought it right not to loſe the opportunity which offered of ſending him to the hoſpital. His caſe case had often been shown, while on board, to several medical men, who, not informed of its peculiarity, looked on it with indifference—as a mere nothing. "Oh! that will soon be well," till they were informed of its history, which it required no small share of confidence to believe. Its usual appearance was indeed so trifling, that without such information, those in power could not have adjudged him an object for the hospital, nor the gentlemen of that establishment admitted him, had not his case been stated. I believe then his cure, with the assistance of good diet, and the privation of the inconveniencies (to say the least of them) of a sea life, was thought to be no difficult matter, and at first excited little more attention than a simple case, till ocular demonstration impressed on their minds the singularity both of the case and its cure.

